# Inferior prosthetic hip dislocation requiring revision: A case report

**DOI:** 10.1016/j.ijscr.2025.111710

**Published:** 2025-07-22

**Authors:** Alexander Burbelo, William Stone, Liam Cleary, Matthew Bullock, Alexander Caughran

**Affiliations:** a Marshall University, Joan C. Edwards School of Medicine, 1600 Medical Center Dr, 25701 Huntington, WV, USA; b Department of Orthopaedic Surgery, Marshall University, Joan C. Edwards School of Medicine, 1600 Medical Center Dr, 25701 Huntington, WV, USA

**Keywords:** Inferior hip dislocation, Total hip arthroplasty, Hip dislocation revision, Prosthetic hip dislocation, case report

## Abstract

**Introduction and importance:**

Total hip arthroplasty (THA) is a highly successful orthopaedic procedure performed for various indications. While rare, complications such as dislocation do occur. We present the case of a 59-year-old female who had a late inferior hip dislocation after a traumatic fall.

**Presentation of case:**

The patient who had a recent THA for femoral neck fracture experienced an incarcerated inferior prosthetic dislocation following mechanical fall. Revision surgical intervention yielded satisfactory stability, but patient noncompliance led to a subsequent dislocation, requiring another revision surgery. Despite failed attempts at closed reduction, a constrained acetabular liner was successfully implemented, resulting in a stable THA.

**Clinical discussion:**

This case underscores the complexities of managing inferior hip dislocations, particularly the importance of patient adherence to post-operative care and the need for tailored surgical approaches.

**Conclusion:**

Further investigation into long-term outcomes associated with constrained liners in revision THA is warranted, as well as strategies to enhance patient compliance to mitigate complications.

## Highlights

• Inferior prosthetic hip dislocation is rare after THA, with few cases reported in the current literature.
• Closed reduction failed due to femoral head entrapment and acetabular fracture, requiring open surgery.
• Recurrent prosthetic dislocation was treated with revision THA using a constrained liner for stability.
• This case shows the importance of close post-op follow-up to prevent and manage prosthetic complications.
• Constrained liners may help manage prosthetic instability in high-risk patients after failed THA revision.

## 1 Introduction

Total hip replacement is one of the most successful procedures in orthopedics [1]. While complications are rare, dislocation is a well described complication after hip replacement. Dislocation rates vary from 2 to 10 % [2], the majority of which occur shortly after surgery [3]. Surgical approach can be a contributing factor as the violation of the native tissues can weaken the hip leading to dislocation. Patient-specific risk factors for dislocation of hip prostheses include advanced age and neurologic disease, while operation-specific risk factors include suboptimal implant position and insufficient soft-tissue tension [2].

Inferior dislocation of the native hip (luxatio erecta femoris) is the rarest type of hip dislocation, representing 2–5 % of all hip dislocations [4]. Several papers have detailed native inferior hip dislocation, which primarily occur due to motor vehicle accidents and commonly present with accompanying femoral head or neck fractures [4,5].

The direction of the dislocation provides clarity to the mechanism behind the injury. Superior dislocation is caused by trauma to a hip resulting in extension, abduction, and external rotation, while inferior hip dislocation is caused by trauma to a flexed, abducted, and externally rotated hip [6]. To our knowledge, no prior description of an inferior prosthetic hip dislocation has been published. The objective of this case report is to describe this rare presentation and associated management. This work has been reported in line with the SCARE criteria [7].

## 2 Presentation of case

**Fig. 1:**
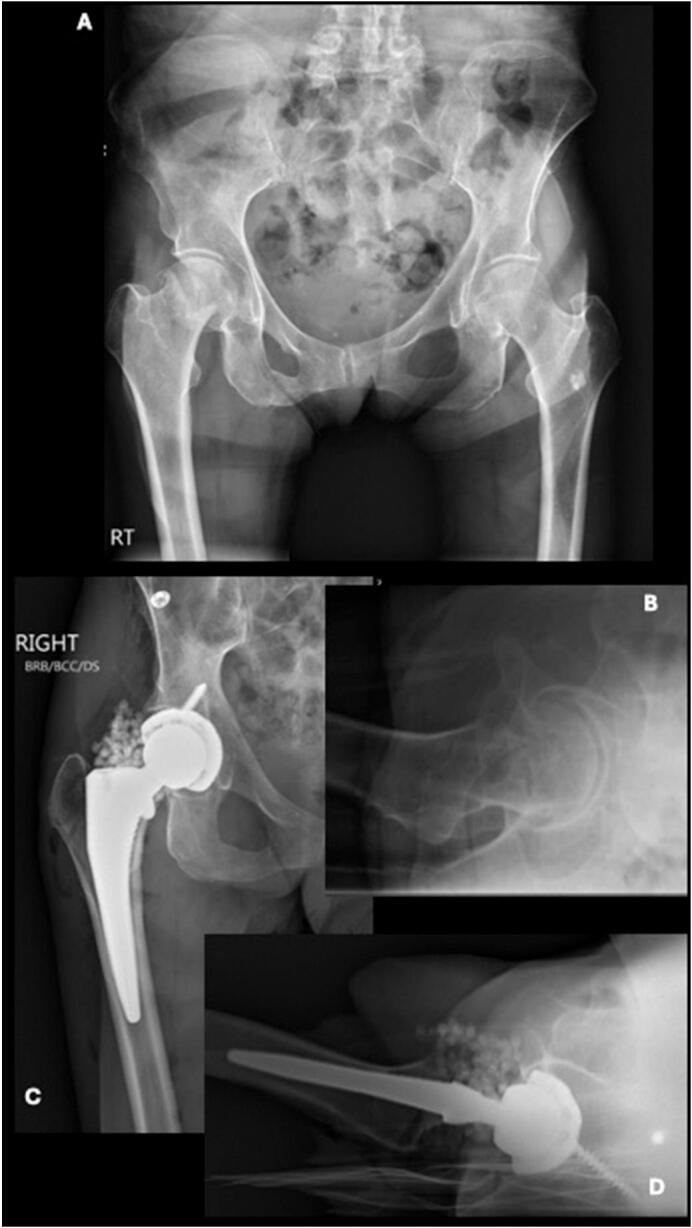
AP (A) and lateral (B) radiographic images of right hip showing displaced right subcapital femoral neck fracture. AP (C) and lateral (D) radiographic images of right hip status post right total hip arthroplasty with placement of antibiotic beads. The beads were used for infection prophylaxis in a high-risk patient, as is standard practice at our institution, particularly in the context of revision surgery when the risk of infection is elevated.

A 59-year-old female with a BMI of 16.05 and a history of substance abuse, osteoporosis, anxiety, depression, and lupus initially presented August 2022, after sustaining a femoral neck fracture after an unwitnessed fall (Fig.1A, B). She successfully underwent a direct anterior THA without complication. Unfortunately, the patient did not present for scheduled follow up.

**Fig. 2:**
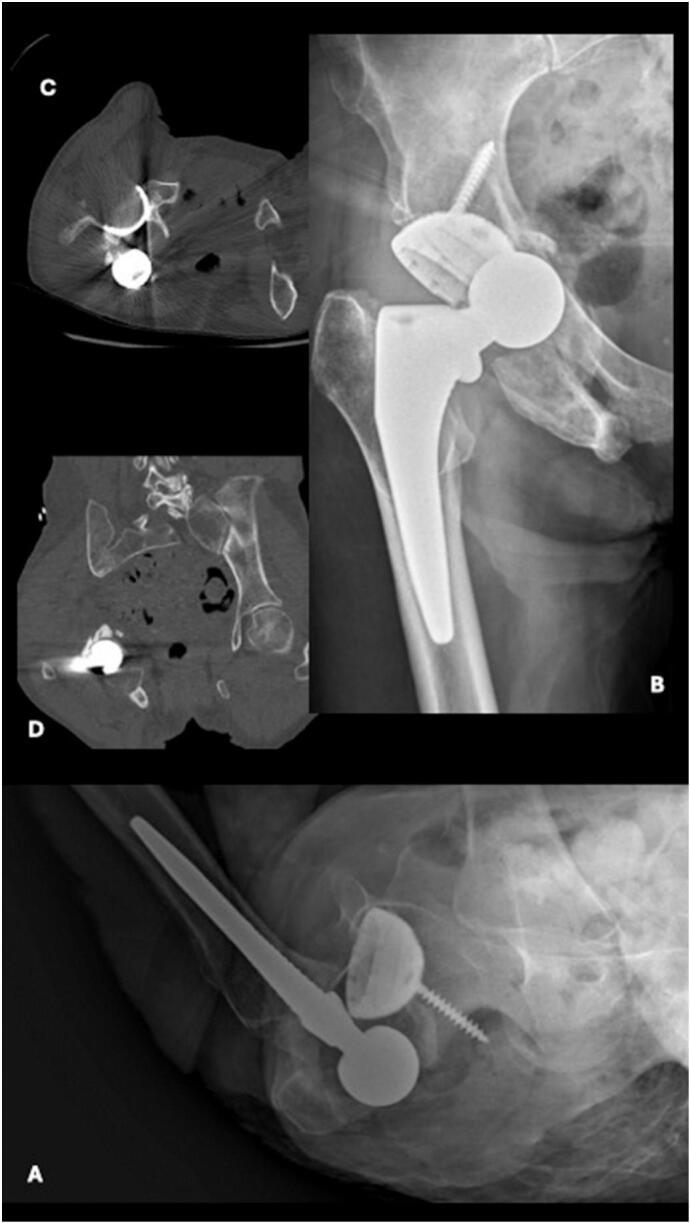
AP (A) and lateral (B) radiographic images of right hip showing right total hip arthroplasty with inferomedial dislocation and associated ischial and inferior pubic ramus fractures. Axial (C) and Coronal (D) CT images of right hip showing right total hip arthroplasty with inferomedial dislocation.

Ten months later, the patient presented to the emergency department ambulating with a walker with right hip pain and swelling (Fig. 1C, D). Although the extremity was neurovascularly intact, the patient was unable to move the hip secondary to pain. The patient reports falling from standing height two days prior and was unable to bear weight on the extremity. Radiographs demonstrated a posteroinferior dislocated femoral component along with a fracture of the inferior ischium and pubic ramus (Fig. 2A, B). A subsequent computed tomography (CT) scan was obtained to assess hip implant orientation, further evaluate the comminuted pelvic fracture, and rule out a proximal femur fracture (Fig. 2C, D). A closed reduction under conscious sedation in the emergency room was attempted but was unsuccessful.

**Fig. 3:**
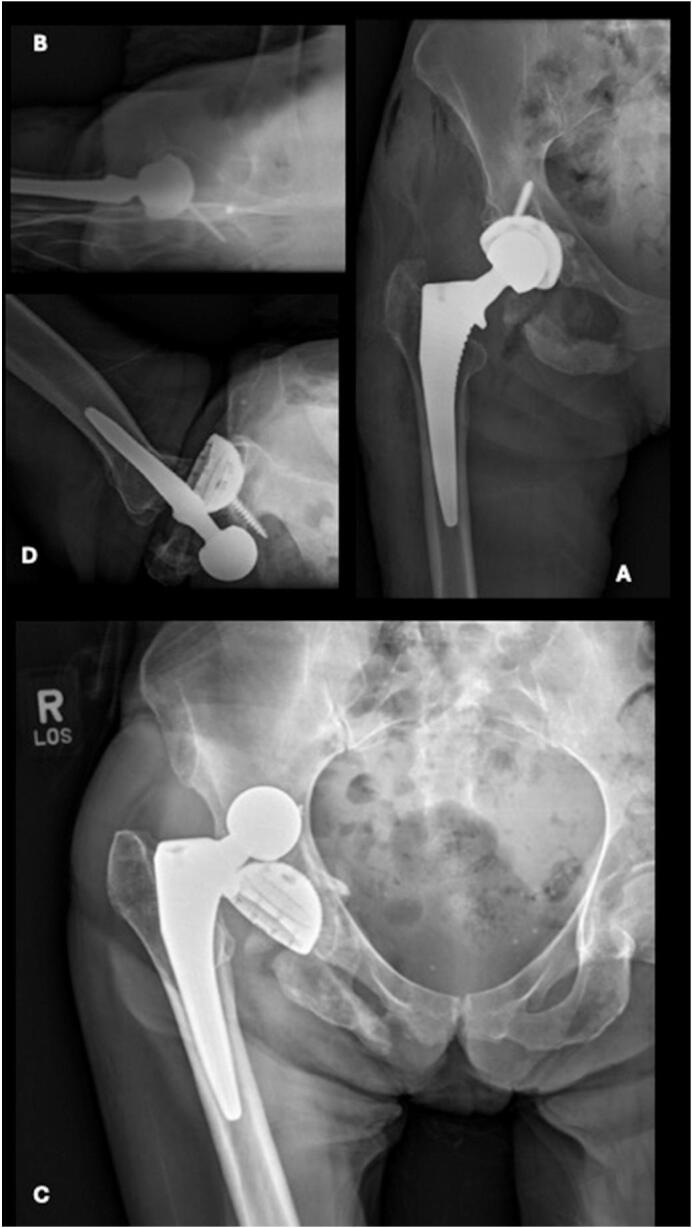
AP (A) and lateral (B) radiographic images of right hip showing concentrically reduced right total hip arthroplasty. AP (C) and lateral (D) radiographic images of right hip status post right total hip arthroplasty with posterior and superior dislocation.

Two days later, the patient was taken to operating theater and underwent another attempt at a closed reduction under anesthesia which was again unsuccessful. The decision was made to perform an open reduction through the prior anterior approach. After exposure, the prosthetic femoral head was noted to be incarcerated beneath the inferior portion of the acetabular component (Fig. 3A, B). A bone hook was used around the femoral neck to gently pull lateral and superior as traction was pulled on the leg to reduce the prosthetic hip dislocation. There was notable disruption of the surrounding anterior and medial hip joint capsule and adductor musculature but no over evidence of abductor muscle injury. The inferior ramus and ischial fractures were managed non-operatively.

**Fig. 4:**
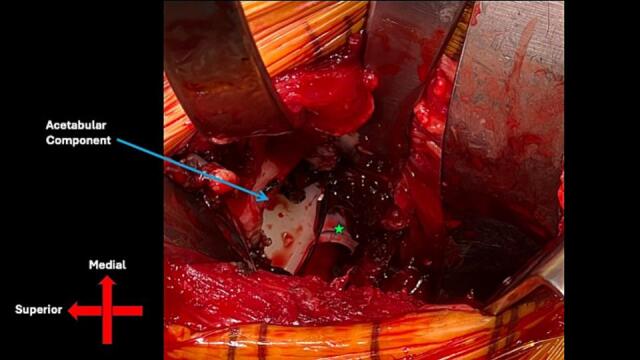
Intraoperative direct anterior approach from right hip revision surgery. Femoral head (green star) is located inferior and medial to acetabular component (blue arrow). (For interpretation of the references to colour in this figure legend, the reader is referred to the web version of this article.)

The patient had an uneventful post operative course and was subsequently discharged home three days after surgery. The patient missed her initial follow-up appointment. She presented two months after revision surgery with continued pain in the right hip and inability to bear weight. Radiographs now demonstrated a superoposterior prosthetic dislocation (Fig. 3C, D). The direction of this dislocation is consistent with prior soft tissue disruption from the earlier anteroinferior dislocation, which likely compromised the hip capsule and contributed to global instability of the prosthetic hip joint. The leg remained neurovascularly intact, and the prior pelvic fractures demonstrated appropriate healing. She was taken to the operating room the next day, where it was determined the chronic hip instability would be best managed with placement of a constrained liner, given the now global instability of the hip (Fig. 4).

**Fig. 5:**
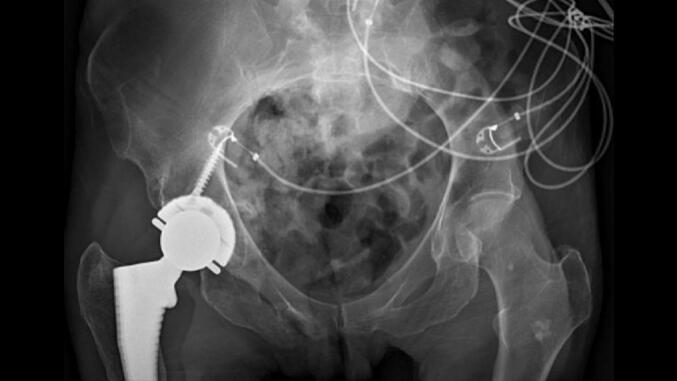
AP radiographic image of pelvis showing right revision total hip arthroplasty with constrained liner.

The patient was discharged and returned nine days after surgery ambulating with a walker. She once again was lost to follow up but had several visits to the emergency department for unrelated issues at which time imaging showed a concentric hip joint and successful healing of the fractures (Fig. 5). Using digital templating software, we calculated the combined anteversion of the hip components to be 28 degrees, which falls within the commonly accepted range of 25–40 degrees, as reported by multiple studies [8].

## 3 Discussion

To our knowledge, inferior prosthetic hip dislocation is a serious complication that has not been previously described. For both native and prosthetic hip dislocations, posterior and anterior hip dislocations are much more frequently observed [3]. Patients with a smaller center-edge angle, particularly those with a shallow acetabulum, were found to have higher risk of dislocation after THA [9]. This patient demonstrated satisfactory stability and range of motion following her THA but failed to present for appropriate follow-up post-operatively, which limited our ability to effectively manage her condition and possibly prevent subsequent complications. Substance use disorder, anemia, and psychiatric disease have all been linked to an increased risk of hip dislocation, which may have contributed to the patient's presentation [2].

Other cases of native inferior hip dislocations have been reported, especially after a traumatic fall [5] or motor vehicle collision [10]. There are two distinct mechanisms of inferior hip dislocations: obturator and ischial [10]. In the obturator type, force is exerted on an abducted hip, which is then flexed and externally rotated to dislocate the femoral head anteriorly and inferiorly to the obturator foramen [10]. In the ischial type, force is exerted on a flexed hip and knee, with the femur in extreme flexion, causing the femoral head to dislocate inferiorly and lie adjacent to the ischium [10].

We hypothesize that this patient had a mechanism of injury similar to an ischial dislocation. As the patient was a poor historian, the true mechanism will not ever be known. When evaluating patients with prosthetic instability, it is important to assess the femoral component for appropriate leg length and offset, while the acetabular component should have for appropriate inclination and version. Intraoperative assessment should evaluate for soft tissue or bony impingement as well as joint laxity. Should there be any issues identified, they should be addressed at the time of the revision surgery. In this case, the leg lengths and offset and component position appeared satisfactory, therefore no additional changes to the components were necessary. During the time of the second revision, it was felt that there was a joint capsule deficiency therefore a constrained liner was utilized. In this case, the soft tissue constraints, particularly the hip capsule, were disrupted during the initial dislocation, potentially predisposing the joint to subsequent bony or component impingement and dislocation.

The inferior ramus serves as the origin for several muscles, including obturator externus, adductor magnus, quadratus femoris, adductor longus, adductor brevis, and gracilis [11]. A fracture in this area could impair these muscles, rendering them ineffective against the unopposed pull of the hip abductors. One possible cause for such a fracture is a forceful impact with the ground, which could cause the prosthetic head to fracture the inferior ramus, potentially incarcerating the head inferior to the acetabular component. In this scenario, the iliopsoas muscle would reflexively contract, generating a flexion moment at the hip. However, motion would not occur, as the head is positioned medial and inferior to the center of rotation of the hip joint.

When closed reduction attempts are unsuccessful and global instability is present, a constrained acetabular liner may be contemplated to address post-operative instability [12]. Short term outcomes of constrained liners have been shown to provide robust stability in high-risk patients needing revision THA, with one study showing no dislocations at an average follow-up of 2.4 years post-THA [13]. Constrained liners are usually reserved for older, lower demand patients, and those with compliance issues.

Despite the challenges associated with her case, the patient maintained a functional hip following her revision surgery with the constrained liner. Constrained liners have been shown to have high rates of aseptic failure at intermediate follow-up post revision THA, but the results were not significantly different compared to dual-mobility liners [14]. Dual-mobility liners and constrained liners with 36 mm heads appeared to have comparable intermediate-term survival compared to standard THA, while smaller constrained liners (<36 mm) are associated with a higher failure rate [15]. Furthermore, severely underweight patients such as ours are more likely to undergo revision THA due to dislocation, which may provide another contributing factor to this patient's condition [16].

## 4 Conclusion

This case highlights the success of revision THA with a constrained acetabular liner in the management of a complex inferior dislocation that was refractory to closed reduction. Nevertheless, this case also illustrates the importance of assessing component position, as well as surrounding soft tissue function to help prevent impingement and subsequent dislocation during revision surgery. Further research is needed to investigate the long-term follow-up associated with rare dislocations such as these, as well as strategies to improve patient compliance.

## Consent

Written informed consent was obtained from the patient for publication of this case report and accompanying images. A copy of the written consent is available for review by the Editor-in-Chief of this journal on request.

## Ethical approval

Our institution requires no ethics approval for case reports reporting on a single case, but we did have patient consent for this manuscript.

## Funding

We have no sources of funding to disclose.

## Author contribution

**Alexander Burbelo:** Writing – review & editing, Writing – original draft, Conceptualization, Investigation. William Stone: Writing – original draft. **Liam Cleary:** Writing – original draft, Writing - review & editing. Matthew Bullock: Writing – review & editing, Supervision, Project Administration, Methodology, Investigation, Conceptualization. **Alexander Caughran:** Writing – review & editing, Supervision, Project administration, Investigation, Methodology, Conceptualization. Guarantor: Dr. Matthew Bullock.

## Guarantor

Dr. Matthew Bullock.

## Research registration number

N/A.

## Conflict of interest statement

All authors declare that they have no conflicts of interest.
